# Biofilm formation associated with calcium phosphate coating on implant metals

**DOI:** 10.1007/s10856-026-07053-y

**Published:** 2026-04-21

**Authors:** David Mrosek, Nataniel Białas, Aileen Winter, Oleg Prymak, Kateryna Loza, Matthias Epple

**Affiliations:** Inorganic Chemistry and Center for Nanointegration Duisburg-Essen (CENIDE), University of Duisburg-Essen, Essen, Germany

## Abstract

Three clinically relevant implant metals, i.e., stainless steel (SS316L), pure titanium (grade 4), and the titanium alloy Ti6Al4V (grade 5), were coated with octacalcium phosphate from supersaturated aqueous calcium phosphate solution. Calcium phosphate coatings are frequently applied to enhance the osteoconductivity of metal implants. However, this leads to a higher surface roughness that increases the risk for bacterial adhesion and biofilm formation. The bacterial species *Escherichia coli* (Gram-negative rods) and *Staphylococcus xylosus* (Gram-positive cocci) were seeded on the bare metals and the calcium phosphate-coated metals, respectively, and cultivated for up to 72 h to assess the biofilm formation. The efficiency of biofilm production by bacteria was evaluated by the crystal violet assay, scanning electron microscopy, and confocal microscopy. The growth of *S. xylosus* was always strong, with and without calcium phosphate coating, whereas *E. coli* proliferated better on calcium phosphate-coated metals. Both bacterial species colonized cavities within the porous calcium phosphate coating as indicated by scanning electron microscopy. The metabolic activity of *S. xylosus* caused a pH drop to 5.5 that led to corrosion of the calcium phosphate layer by acidic dissolution. In contrast, *E. coli* led to an increase in pH to about 8.9 that did not affect the coating. Osteoblast-like MG-63 cells adhered and proliferated well on both coated and uncoated metals, underscoring the good osteocompatibility before and after coating.

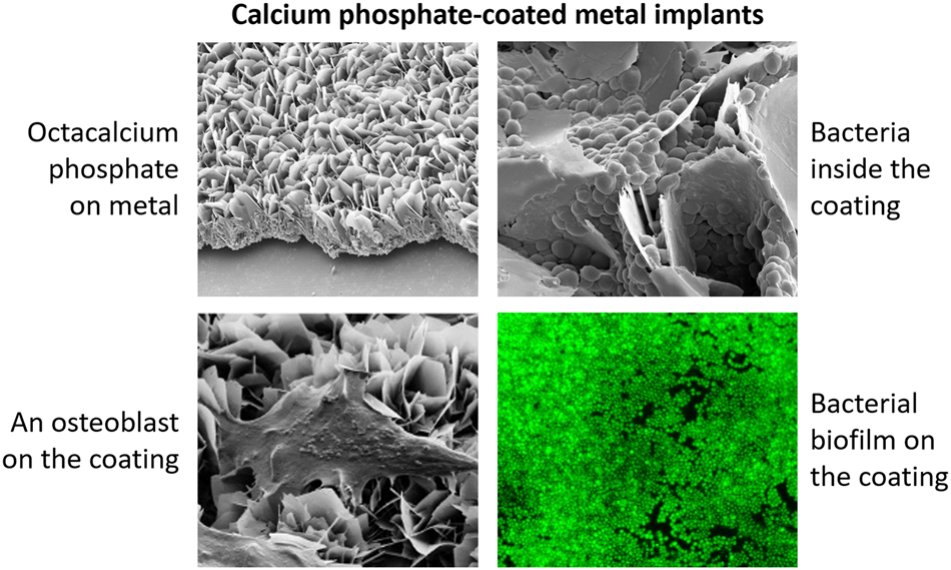

## Introduction

Microbial infections are a persistent risk factor after the implantation of metallic implants like endoprostheses or dental implants [1–8]. The formation of bacterial biofilms on the implant surface, which are almost impossible to treat by conventional therapeutic methods (e.g., with antibiotics), usually requires revision and re-implantation after mechanical treatment (i.e., breaking up the biofilm matrix) and chemical cleaning (i.e., by antibiotics, disinfectants) to clear the implantation site from bacteria [9, 10]. If untreated, the local accumulation of bacteria may lead to severe consequences for the patient, including the transmission of the bacterial infection into the bloodstream (bacteremia) and the development of a life-threatening sepsis [11]. The difficulty of biofilm eradication results from the fact that biofilms are multicellular microbial networks embedded in a dense matrix composed of extracellular polymeric substances, i.e., EPS (mainly polysaccharides) [12, 13]. The growth inside a biofilm matrix often makes bacterial cells insensitive to antibiotics due to the physical barrier between the drug and the immobilized cell [14].

Implants like endoprostheses or dental implants usually consist of biocompatible metals like titanium, titanium alloys, and stainless steel [15]. To enhance their osteoconductivity and bioactivity, they can be coated with calcium phosphate, which represents the inorganic part of mammalian bone [16–18]. This also increases the surface roughness, which facilitates cell adhesion and bone on-growth [19–21]. Different coating methods are used, e.g., high-temperature plasma spraying [22], coating from supersaturated solution [23], electrophoretic deposition of nanoparticles [24], and magnetron RF plasma sputtering [25]. Unfortunately, an increased surface roughness usually promotes the attachment of bacteria and biofilm formation [26, 27]. Thus, increasing the surface roughness of an implant by coating it with calcium phosphate is a double-edged sword in terms of infection prevention.

Here we show how two different bacterial species, i.e., *Escherichia coli* (DSM 5698, Gram-negative rods) and *Staphylococcus xylosus* (DSM 6179, Gram-positive cocci) as models for pathogenic bacteria were cultivated on three typical implant materials, i.e., stainless steel SS316L, pure titanium (grade 4), and the titanium alloy Ti6Al4V (grade 5). The materials were studied as plain polished metals and also after coating with calcium phosphate from a supersaturated calcium phosphate solution. In addition, the osteocompatibility of the materials was confirmed by seeding MG-63 osteoblast-like cells on their surface.

## Materials and methods

### Reagents

Ultrapure water (Purelab ultra instrument from ELGA) was used for all syntheses and preparations. For experiments with cells and bacteria, it was also autoclaved for sterilization. All experiments with cells and bacteria were performed with sterile instruments and labware (Sarstedt, Germany).

Liquid LB medium (Lennox; 20 g L^−1^) and CASO bouillon (Trypticase Soy Broth; TSB; 30 g L^−1^) supplemented with yeast extract (3 g L^−1^) were used to cultivate *E*. *coli* and *S*. *xylosus*, respectively. For the preparation of solid media, LB medium and CASO bouillon were additionally supplemented with agar-agar Kobe I (15 g L^−1^). All reagents for the preparation of the growth media were obtained from Carl Roth (Germany). Other reagents were crystal violet (0.1% in double-distilled water; Merck, Germany), ethanol (p.a., ≥99.5%; VWR, Germany), Dulbecco’s phosphate-buffered saline (DPBS, pH 7.4; Gibco, Germany), and paraformaldehyde (3.7%; Merck). Cell culture media (DMEM and Ham’s F12 medium) and supplements (fetal calf serum, penicillin-streptomycin, phosphate-buffered saline and GlutaMax™) were obtained from Gibco. A cell imaging kit (Calcein-AM) was obtained from Thermo Fisher Scientific (USA). The BacLight™ bacterial imaging kit was obtained from Invitrogen (USA).

Titanium (grade 4), Ti6Al4V (grade 5), and stainless steel 316 L sheets (all obtained from Goodfellow, Germany) were cut into pieces of 10 × 10 mm^2^. The thickness was 1 mm for titanium and Ti6Al4V and 3 mm for stainless steel. Potassium hydroxide (Thermo Fisher Scientific) and hydrogen peroxide (Carl Roth) were used to etch the metal surfaces. For calcium phosphate coating, calcium lactate pentahydrate (Merck), potassium dihydrogen phosphate (Merck), and potassium hydroxide (Thermo Fisher Scientific) were used.

### Methods and instruments for characterization

Scanning electron microscopy (SEM) was performed with an Apreo S LoVac instrument from Thermo Fisher Scientific, combined with energy-dispersive X-ray spectroscopy (EDX) by an UltraDry silicon drift X-ray detector (Thermo Fisher Scientific). Prior to SEM analysis, all samples were sputter-coated with Pd/Au.

TEM investigations were performed with an FEI Titan microscope (Thermo Fisher Scientific) equipped with a Cs-probe corrector (CEOS, Germany) and operated at an accelerating voltage of 300 kV [28]. For TEM analysis, the calcium phosphate crystals were mechanically scraped off a coated titanium substrate. The collected crystals were dispersed in ethanol, and a small droplet of the suspension was deposited onto a carbon-coated copper TEM grid. The grid was dried under ambient conditions and subsequently used for TEM imaging. FFTs of the HRTEM images were analyzed with the CrystBox software [29]. and the resulting diffraction patterns were indexed by comparison with reference data from the ICDD database.

X-ray powder diffraction (XRD) was carried out with a Bruker D8 Advance powder diffractometer in Bragg-Brentano reflection mode with Cu Kα radiation (*λ* = 1.54 Å; *U* = 40 kV, *I* = 40 mA). The metal substrates were mounted on a sample holder and measured from 5 to 90 °2θ with a step size of 0.01 °2θ (total measuring time 3.5 h). Additionally, the samples were investigated by grazing incidence diffraction (GIXRD) on a Panalytical Empyrean instrument with Cu Kα radiation (*λ* = 1.54 Å; *U* = 40 kV, *I* = 40 mA). GIXRD was carried out with an incident beam angle of *Ψ* = 1.0° with respect to the sample surface and at 2θ from 1° to 90° with a step size of 0.02 °2θ (total measuring time 2.5 h). Qualitative phase analysis was performed by Diffrac. Suite EVA V7.1 from Bruker with the patterns of metallic substrates, i.e., hexagonal α-Ti (#44-1294) and cubic Fe (#52-0513), as well as of octacalcium phosphate coating OCP (#79-0423). Quantitative Rietveld refinement was performed with the Bruker software TOPAS 7.0 to calculate the lattice parameters and the average crystallite size *CS*. For this, the instrumental correction was determined with a LaB_6_ standard powder sample from NIST (SRM 660b).

### Coating of metals with calcium phosphate from a supersaturated calcium phosphate solution

We followed the procedure reported in ref. [30]. The metal samples were ultrasonically cleaned in ethanol and acetone for 5 min each and dried in air. For surface activation, each plate was etched in a mixture of 10 mL 5 M aqueous potassium hydroxide solution and 2 mL 30% aqueous hydrogen peroxide solution for 20 min at 115 °C in a drying cabinet. To remove any formed titania flakes, the flask was shaken every 5 min. After etching, the plates were thoroughly rinsed with water and placed into a supersaturated calcium phosphate solution.

The supersaturated calcium phosphate solution was prepared as follows: First, 90 mL of distilled water was put into a round-bottom flask. 2 mL of 0.10 M potassium dihydrogen phosphate solution was added, and the pH was adjusted to ~7.25 with 0.1 M potassium hydroxide solution. Then, 1.5 mL of 0.12 M calcium lactate solution and 3.75 mL water were added, and the flask was thoroughly shaken. Finally, 1.5 mL of 0.12 M calcium lactate solution was added, and the solution was thoroughly shaken again. The coating with calcium phosphate was performed for 36 h at room temperature without stirring. After the coating process, the plates were rinsed thoroughly with water and dried in air.

### Bacteria and eukaryotic cells

We used the Gram-negative species *Escherichia coli* (DSM 5698, BSL-1 class) and the Gram-positive species *Staphylococcus xylosus* (DSM 6179, BSL-1 class) [31]. All bacteria were obtained from the German Collection of Microorganisms and Cell Cultures (DSMZ, Braunschweig, Germany) as freeze-dried cultures. The osteoblast-like adherent MG-63 cell line (ATCC-CRL-1427, BSL-1 class) was used as a model for cell-material interactions in terms of biocompatibility [32].

### Biofilm formation by bacteria and crystal violet assay

Bacteria were grown at 37 °C either in an orbital shaker MAXQ 4000 (Thermo Fisher Scientific) at 130 rpm or on solid agar plates in a Heratherm Compact incubator (Thermo Fisher Scientific). The bacterial growth during the preparation of liquid cultures for biofilm formation experiments was monitored with a Biowave CO8000 turbidity meter (WPA, United Kingdom). Colony-forming units (CFU) on solid agar plates were counted with an SC6+ digital colony counter (Stuart, United Kingdom). Bacterial biofilms were prepared on coated and uncoated metal substrates and on ultrathin square glass coverslips as a control (18 × 18 mm^2^; thickness 170 ± 5 µm; Carl Roth).

For studies on biofilm formation, log-phase bacterial cultures were prepared from overnight cultures. Quantification of the bacterial biofilm biomass was performed by the crystal violet assay [33], adapted from Dordet-Frisoni et al. [34]. The sterile medium was inoculated with an overnight culture (2% inoculum, *V*/*V*) and grown until the fresh culture had reached an optical density (OD) of 0.6, measured by turbidimetry at *λ* = 600 nm. Metal samples (with and without calcium phosphate coating) were flame-sterilized, transferred to sterile 12-well plates, and immersed in 3 mL log-phase bacterial cultures for up to 72 h at 37 °C under gentle shaking (50 rpm). No medium exchange was performed during the incubation to permit undisturbed biofilm growth. After incubation, the metal samples with the biofilm on them were carefully transferred to new 12-well plates and gently washed twice with double-distilled water to remove loosely attached and planktonic bacteria and then stained with 0.1% crystal violet solution for 15 min. Following the staining, the dye was aspirated, the biofilms were washed twice with water to remove excess dye and decolorized with absolute ethanol for 15 min. The solubilized dye (released from biofilm-forming bacterial cells) was transferred to 96-well microplates, and the absorbance was measured spectrophotometrically at *λ* = 620 nm with a HiPo MPP-96 microplate photometer (Biosan, Latvia). As controls, samples without bacteria (uncoated and calcium phosphate-coated metals) were incubated with the dye solution to assess the effect of unspecific dye absorption by the substrates.

### pH monitoring in biofilm cultures

For pH monitoring studies, bacterial cells were pelleted with a Heraeus Multifuge X1R centrifuge (Thermo Fisher Scientific). The pH of the growth media during cultivation of bacterial biofilms was measured with an inoLab pH 7110 pH-meter combined with a SenTix^®^ 41 electrode (WTW, Germany). Bacterial cultures for pH monitoring experiments were prepared in the same way as in the studies on biofilm formation by bacteria. The experiments were carried out for up to 72 h at 37 °C under gentle shaking (50 rpm). pH changes were monitored in the presence of an uncoated Ti6Al4V plate in each well (12-well plates). At given time points, the medium was removed from the wells, and the bacterial cells were pelleted. The supernatants were collected, sterile-filtered (0.2 µm; Filtropur S; Sarstedt), and the pH was measured at room temperature. As references, the growth media LB and TSB were measured as well.

### Cultivation of MG-63 osteoblasts on substrates

Prior to cell cultivation, the substrates were sterilized by immersion in 70% ethanol solution (*V*/*V*), followed by two subsequent rinses with water to remove residual ethanol. MG-63 osteoblasts were cultivated in a 1:1 mixture of Ham’s F12 medium and DMEM containing 15% FCS, 2 mM L-glutamine, 100 µg mL^−1^ penicillin, and 100 U mL^−1^ streptomycin at 37 °C and 5% CO_2_ atmosphere for 48 h in a T75 cell culture flask (Sarstedt). Cells were then seeded onto the plates in a 12-well plate (10^5^ cells per well) and further cultivated for 48 h under standard cell culture conditions. Cells were also cultivated on ultrathin square glass coverslips as a control (18 × 18 mm^2^; thickness 170 ± 5 µm; Carl Roth).

### Preparation of biofilms and cells for scanning electron microscopy

The morphological characterization of bacterial biofilms was conducted by scanning electron microscopy. To preserve the mechanically fragile structure of the biofilms, the samples were fixed with 3.7% paraformaldehyde and then gently washed only once with PBS, avoiding serial washing steps. In our experience, multiple washing steps led to structural damage in preliminary experiments, where a partial disruption of the biofilm after drying in an ascending ethanol row due to the applied mechanical force had occurred. Thus, no dehydration of the biofilm samples by ethanol was performed to preserve the biofilm structure as far as possible. Instead, the samples were slowly dried in air at 37 °C for 2 h.

MG-63 cells were fixed with 3.7% glutaraldehyde (Sigma-Aldrich) for 15 min at room temperature, followed by three rinses with PBS. Dehydration was carried out with an ascending ethanol row (20%, 40%, 60%, 80%, and 96%), with each step lasting for 5 min. After the final ethanol step, the samples were incubated in hexamethyldisilazane (HMDS, Merck) for 10 min.

### Confocal laser scanning microscopy

For confocal laser scanning microscopy, samples colonized with *E. coli* and *S. xylosus* were stained with the BacLight™ Bacterial Viability Kit as follows. First, a dye solution of 3.34 mM SYTO-9 in DMSO in autoclaved water was prepared. For staining of bacteria, 3 µL of the dye solution per 1 mL of cell suspension was applied. The dye was added to the bacterial suspension in the 12-well plates with the samples and incubated for 20 min at room temperature. In the next step, the supernatant was removed, and the sample was washed twice with double-distilled water to remove excess dyes. Finally, bacteria were fixed with 3.7% paraformaldehyde for 15 min at 37 °C and washed again twice with double-distilled water. 10 µL of the mounting medium (Invitrogen, Fluoromount-G) was placed on a glass microscopy slide (76 × 26 mm^2^; Thermo Fisher Scientific), and the coverslip was put on the microscopy slide so that the surface with the biofilm sample was carefully immersed, avoiding air bubbles, in the mounting medium.

MG-63 cells were seeded onto metal plates in a 12-well plate (10^5^ cells per well) and cultivated for 48 h under standard cell culture conditions. Next, 120 µL of the staining solution was added to the medium, and the cells were incubated for another 20 min at 37 °C and 5% CO_2_ atmosphere. The metal plates were then transferred to petri dishes (diameter 10 cm) and covered with PBS for investigation by confocal laser scanning microscopy.

The measurements were performed with the confocal microscopes Leica TCS SP8X Falcon, Leica SP8 MP and FLIM (Leica, Germany) at excitation/emission wavelengths of *λ* = 480/500 nm and *λ* = 495/515 nm for SYTO 9 and Calcein-AM, respectively. The images were acquired with an HCX PL Apo 63x/1.4 oil objective and an HC PL APO 40x/1.1 water correction objective, respectively. The Leica TCS SP8X Falcon microscope was used for the analysis of samples on glass substrates in transmission mode, whereas the upright confocal Leica SP8 MP microscope was used for the examination of metal samples in reflection mode.

### Statistics

The crystal violet assay for the determination of the biofilm biomass was performed in three independent biological replicates. For each biological replicate, three measurements were performed and averaged before statistical analysis. The background absorbance originating from ethanol (decolorizer) controls was subtracted from all measurements. The results are presented as mean ± standard deviation. Statistical analysis was performed by two-way analysis of variance (two-way ANOVA) to evaluate the effects of bacterial species and surface type on biofilm biomass, including their interaction. Differences were considered statistically significant at *p* < 0.05. Studies on pH monitoring in biofilm cultures were performed in three independent experiments.

## Results

The following workflow was followed to assess the cell adhesion on coated metals. First, the metals were cleaned and coated with a calcium phosphate layer from a supersaturated solution. Then, the substrates were structurally characterized by electron microscopy and X-ray powder diffraction. Next, osteoblast-like cells were seeded on the substrates to confirm their osteocompatibility. Finally, two bacterial strains, i.e., *E. coli* and *S. xylosus*, were seeded on the coated substrates. The cell adhesion and biofilm formation were assessed by electron microscopy, the crystal violet assay, and confocal microscopy.

**Fig. 1:**
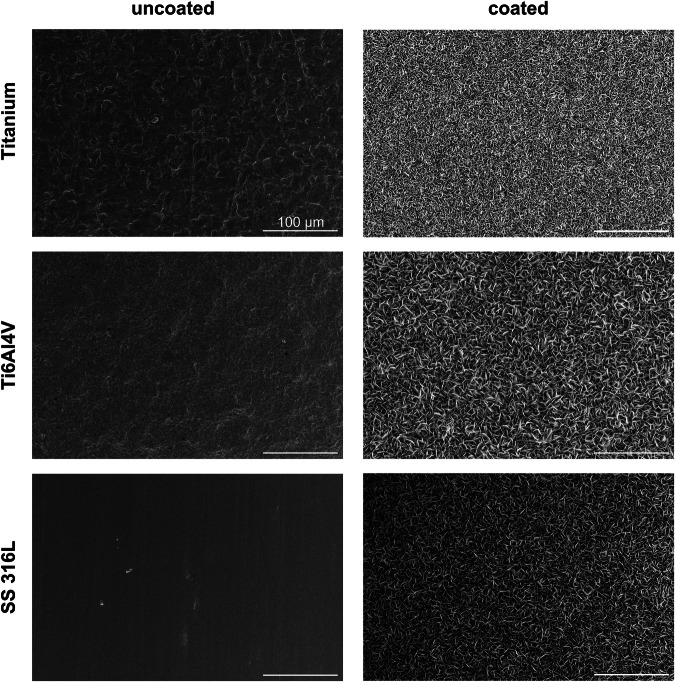
Scanning electron micrographs (top view) of metal plates in the original state (polished; uncoated; **left**) and after etching and coating with calcium phosphate (**right**). All scale bars are 100 µm

**Fig. 2:**
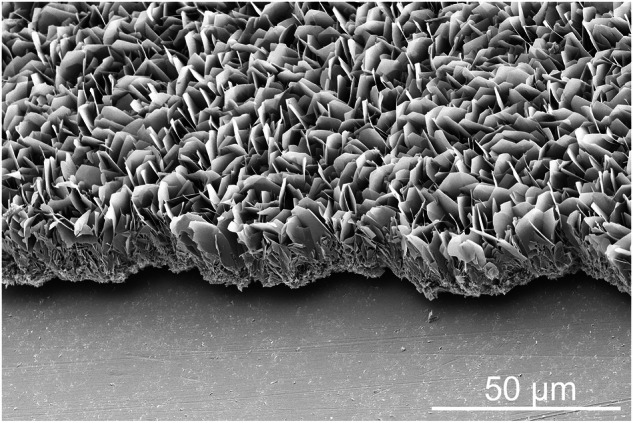
Representative SEM image of a calcium phosphate coating on SS 316 L, seen from the side

The coating of the metal substrates was performed by surface activation (oxidative etching), followed by immersion in a supersaturated calcium phosphate solution. This led to the deposition of plate-like calcium phosphate crystals with a total coating thickness of about 20 µm (Fig. 1). To determine the coating thickness, cross-sectional SEM images of calcium phosphate-coated samples with partially removed coatings (scratching) were taken (Fig. 2). This layer was inherently porous, forming many cavities between the intertwined calcium phosphate platelets.

**Fig. 3:**
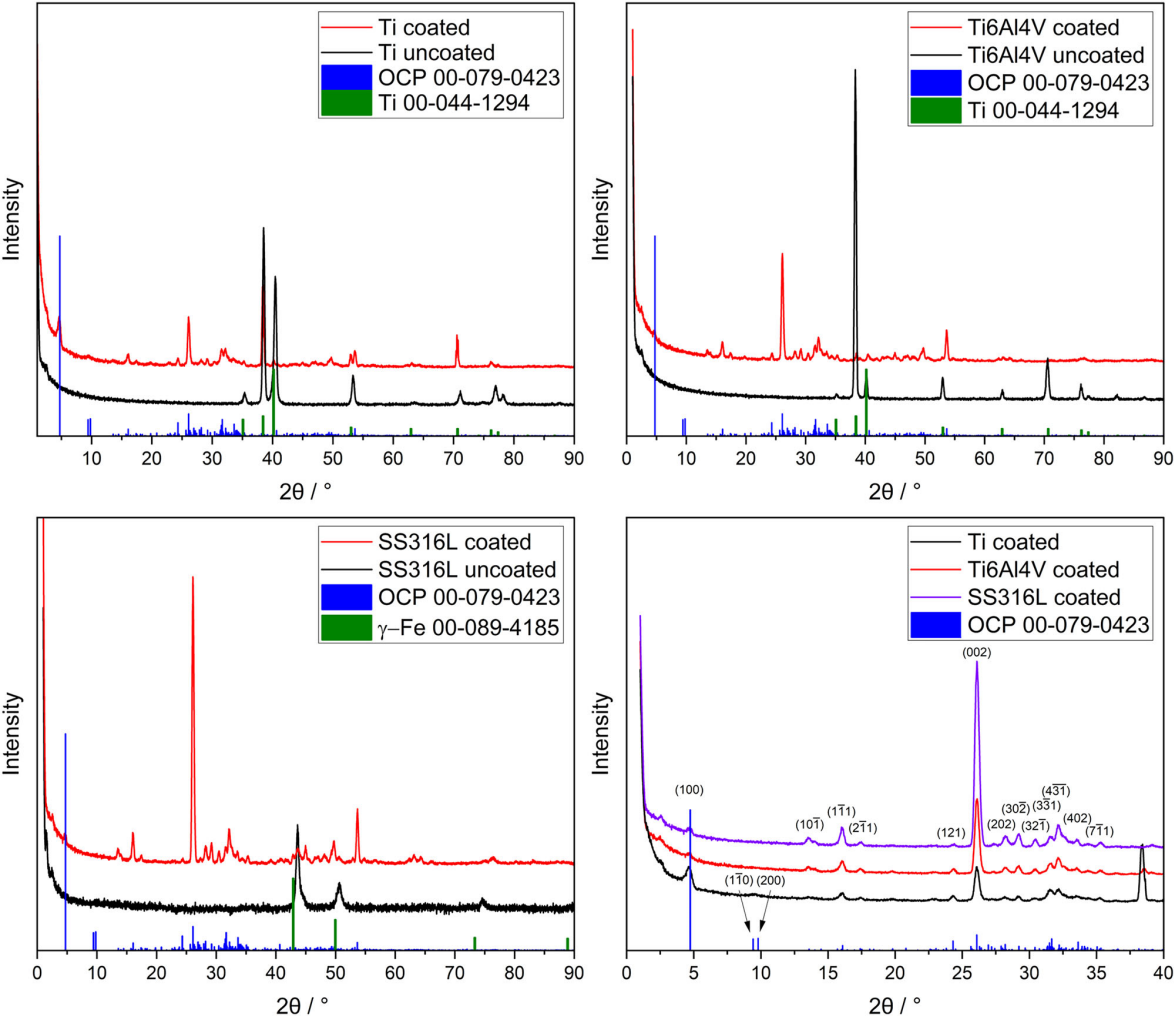
X-ray powder diffraction patterns recorded in the grazing incidence (GIXRD) mode of uncoated and calcium phosphate-coated metal plates. Crystallographically, the coatings consist of octacalcium phosphate. ICDD card numbers are given after the compounds. Miller indices of OCP are given in parentheses

X-ray powder diffraction data of the metal samples before and after coating are displayed in Fig.3. The uncoated substrates, titanium and Ti6Al4V, showed α-titanium, and in the uncoated sample, SS316L, γ-Fe was identified as a crystalline phase. All calcium phosphate-coated samples showed the diffraction peaks of octacalcium phosphate (OCP), Ca_8_H_2_(PO4)_6_ ∙ 5 H_2_O, in good agreement with earlier results where titanium was coated from solution [35–38].

**Fig. 4:**
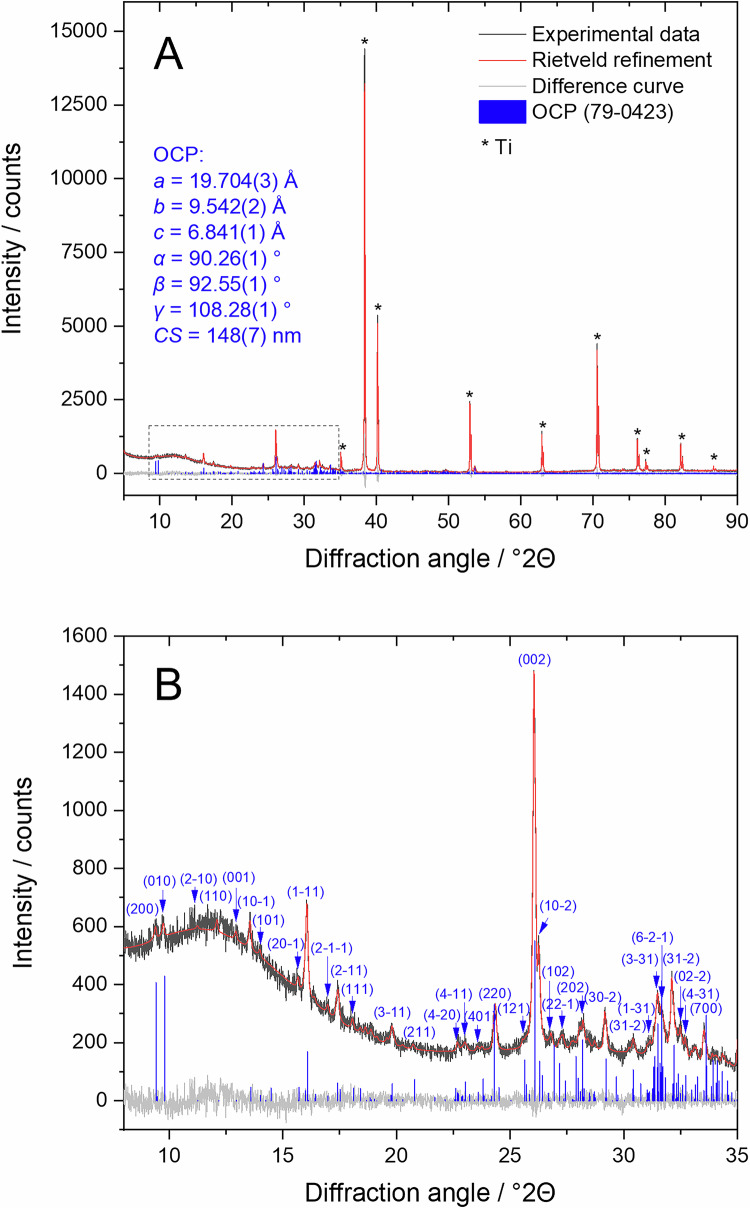
Representative Rietveld refinement of OCP on Ti **A** with a zoomed 2θ region from 8 to 35 ° for a better visualization of the OCP reflection peaks with the Miller indices (**B**). The diffraction peaks of Ti are indicated by asterisks. Miller indices of OCP are given in parentheses. The diffractogram was measured in Bragg-Brentano mode. Rietveld refinement data for calcium phosphate coatings on Ti6Al4V and SS316L are given in the Supplementary Information (Figs. S3 and S4)

Rietveld refinement allowed the clear identification of octacalcium phosphate (OCP), as representatively shown for the OCP coating on titanium (Fig. 4). As a control, the OCP coating was scraped off from six titanium substrates and measured again by XRD as powder on a single crystal silicon sample holder (Fig. S2). The diffraction peaks and lattice parameters agreed well with OCP from the database (#79-0423).

**Fig. 5:**
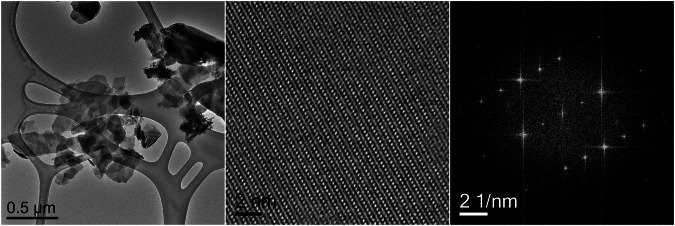
TEM characterization of the detached calcium phosphate coating. Bright-field TEM image (**left**), high-resolution TEM image (**middle**), and corresponding fast Fourier transform (FFT) pattern (**right**), which indicates the [011] zone axis orientation of an OCP crystal

Transmission electron microscopy (TEM) was employed to investigate the structure of the deposited calcium phosphate crystals (Fig. 5). Brightfield TEM showed plate-like particles as the characteristic shape. High-resolution TEM analysis revealed distinct lattice fringes, confirming the crystalline nature of the calcium phosphate. The corresponding fast Fourier transform (FFT) pattern displayed sharp diffraction spots, consistent with crystalline octacalcium phosphate, in this case oriented along the [011] zone axis. The indexed diffraction pattern and the related information are provided in the Supporting Information (Fig. S1).

**Fig. 6:**
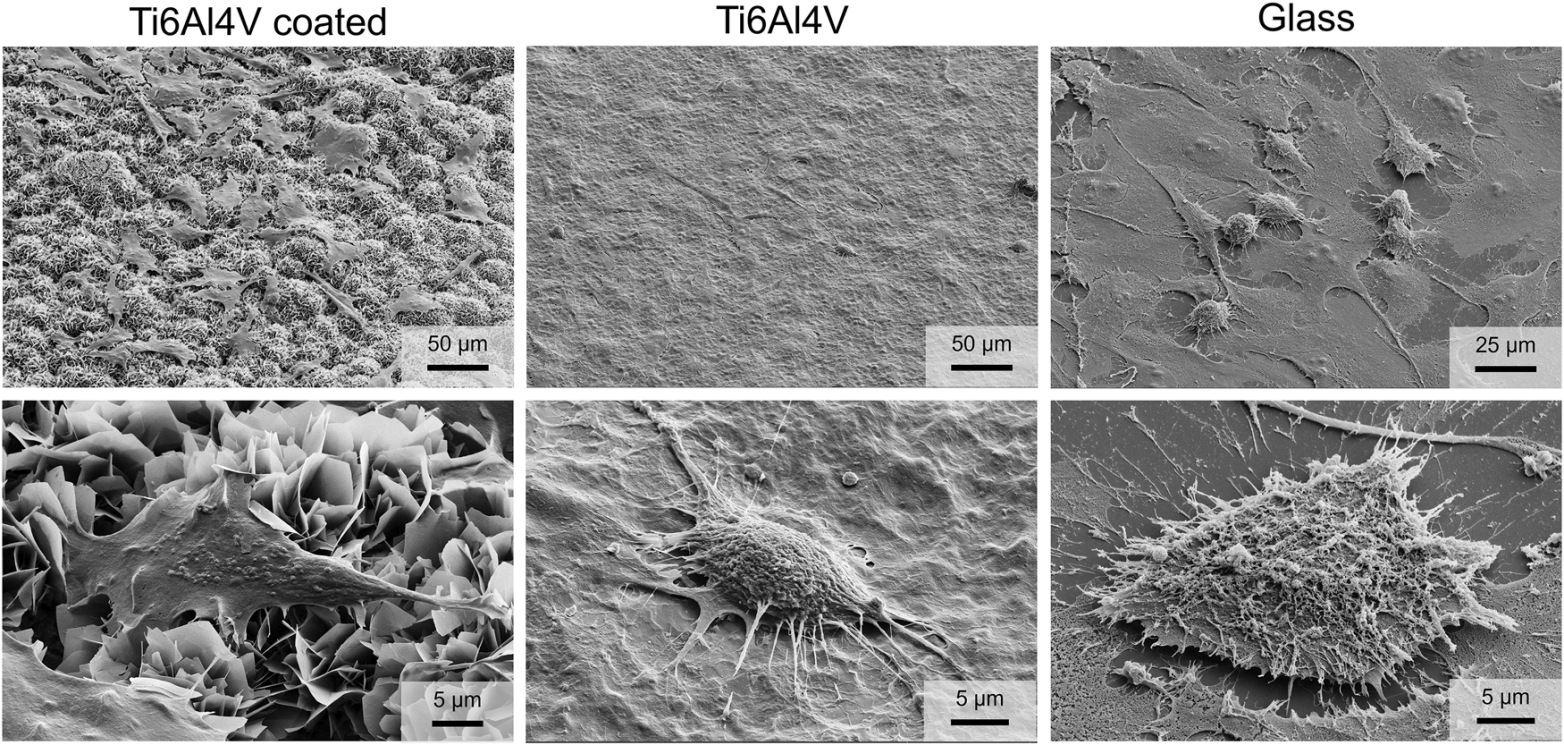
SEM images of MG-63 osteoblast-like cells cultured for 48 h on calcium phosphate-coated Ti6Al4V, on uncoated Ti6Al4V, and on a glass substrate at low (**top row**) and high magnification (**bottom row**)

MG-63 osteoblast-like cells attached very well to uncoated and coated Ti6Al4V. As control, the cells were also cultivated on a glass substrate (Fig. 6). On uncoated Ti6Al4V and glass, the cells formed a dense layer, whereas on calcium phosphate-coated Ti6Al4V, they were spread out to keep in contact with the calcium phosphate platelets.

**Fig. 7:**
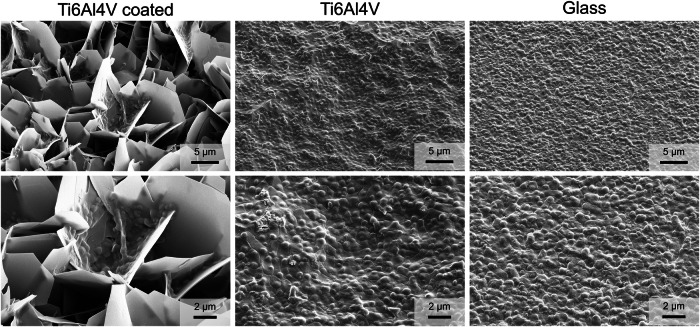
Scanning electron micrographs of *E. coli* biofilms grown for 72 h on calcium phosphate-coated Ti6Al4V, on uncoated Ti6Al4V, and on a glass substrate at low (**top row**) and high magnification (**bottom row**)

**Fig. 8:**
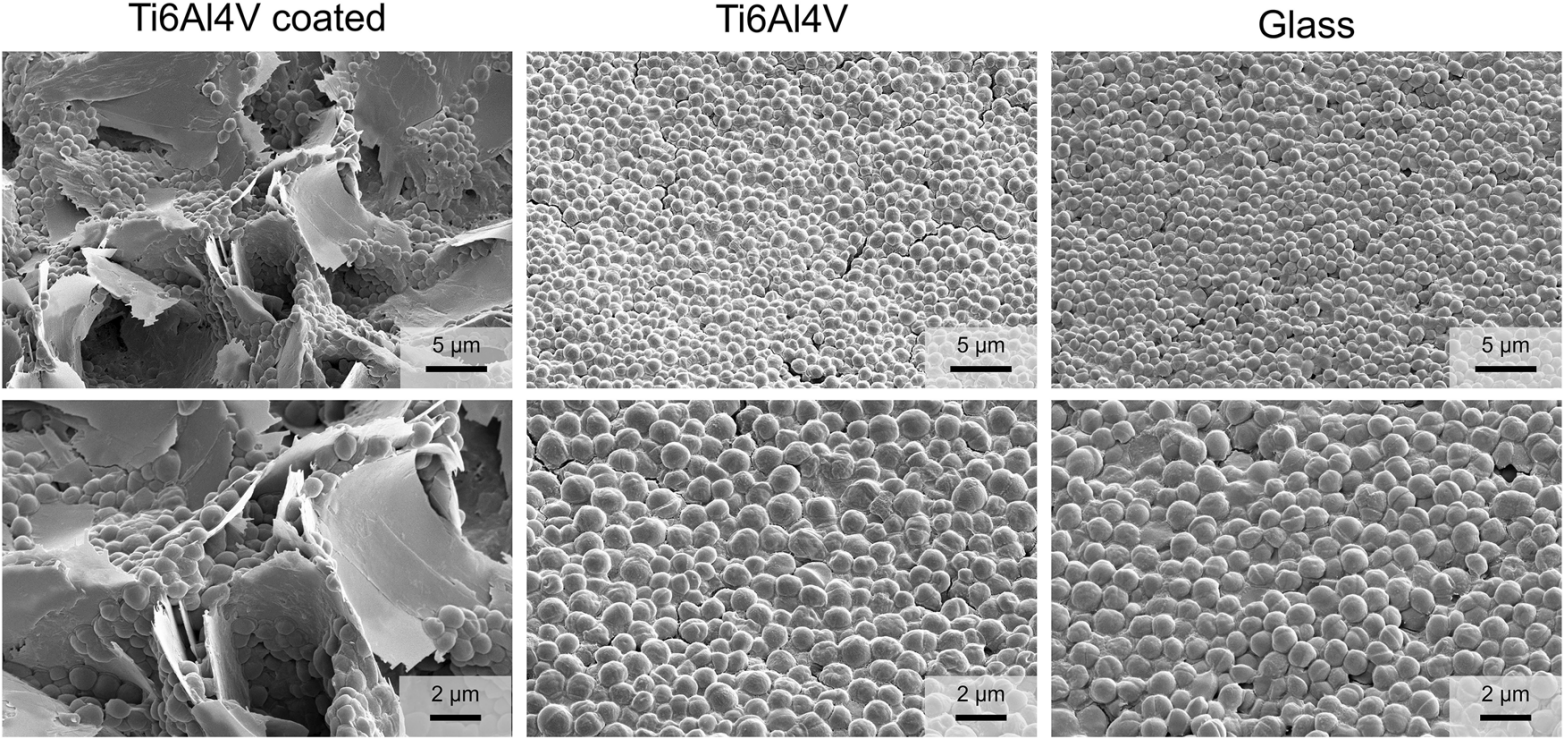
Scanning electron micrographs of *S. xylosus* biofilms grown for 72 h on calcium phosphate-coated Ti6Al4V, on uncoated Ti6Al4V, and on a glass substrate at low (**top row**) and high magnification (**bottom row**)

**Fig. 9:**
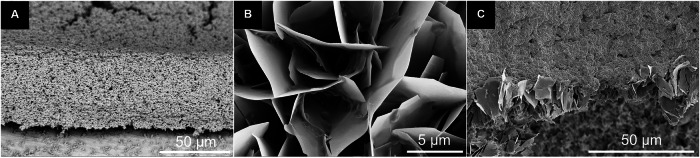
Representative side-view scanning electron micrographs of a *S. xylosus* biofilm on uncoated SS 316 L (**A**), *E. coli* bacteria inside the calcium phosphate coating on Ti6Al4V (**B**), and *S. xylosus* bacteria on calcium phosphate-coated titanium (**C**). All biofilms were grown for 72 h

**Fig. 10:**
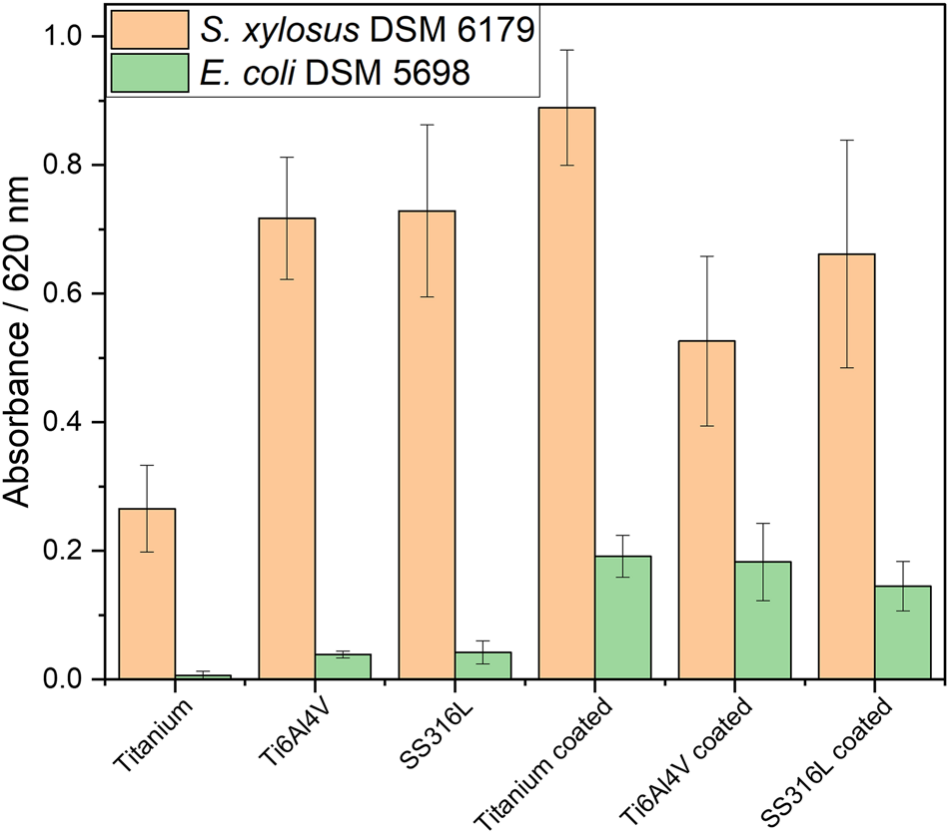
Biofilm production by two bacterial species, i.e., *E*. *coli* and *S*. *xylosus*, grown for 72 h on implant metals with and without calcium phosphate coating. The crystal violet assay gives the total biofilm biomass and does not distinguish between bacterial cells and extracellular matrix components. The values represent mean ± SD of three replicates after subtraction of background absorbance from ethanol controls

Biofilm-forming bacteria were seeded on uncoated and coated Ti6Al4V. *S. xylosus* is a well-known biofilm former and a potent EPS producer [31], whereas *E. coli* has a lower tendency to form biofilms [39]. The adherence of bacteria was visualized by scanning electron microscopy (Figs. 7, 8, and 9). *E*. *coli* formed isolated colonies also inside the calcium phosphate coating, whereas *S*. *xylosus* showed a more extended growth, bridging adjacent calcium phosphate crystals, but also inside the coating. This indicates that the presence of the calcium phosphate coating leads to different biofilm formation for the two bacterial species. The biofilm biomass production was quantitatively assessed by the crystal violet assay (Fig. 10). Statistical analysis using two-way analysis of variance (two-way ANOVA) showed a significant effect of bacterial species on the formed biofilm biomass (*F*(1,24), *p* < 0.0001) and a significant effect of surface type (6 different substrates) on the formed biofilm biomass (*F*(5,24), *p* < 0.0001).

**Fig. 11:**
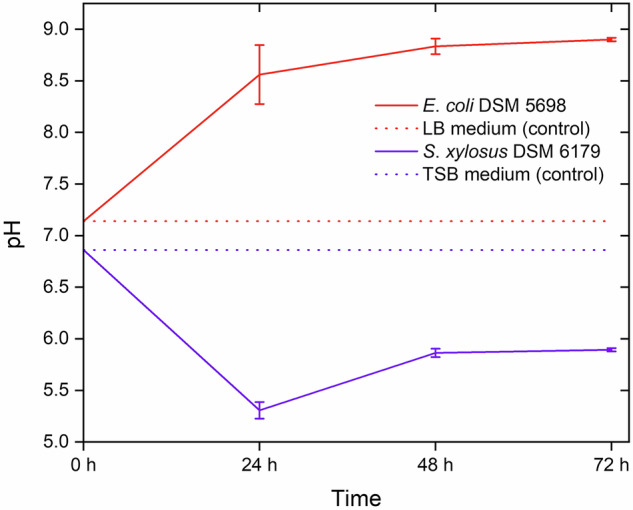
pH of the culture medium during cultivation of bacterial biofilms (*E*. *coli* and *S*. *xylosus*) in the presence of uncoated Ti6Al4V. As a reference, the pH values of the buffered cultivation media (LB, TSB) are shown

We noticed that the edges of the calcium phosphate platelets were more rugged after cultivation of *S. xylosus*, appearing corroded (Fig. 8). Therefore, we monitored the pH of the growth medium during the development of the bacterial biofilms. It turned out that the pH changed significantly for both bacterial species, despite the presence of the buffered cell culture medium. During biofilm development by *E*. *coli*, the pH increased to 8.9, whereas during biofilm development by *S*. *xylosus*, it dropped to about 5.5 (Fig. 11). Of course, such a change in pH will be different in an in vivo situation with different perfusion and buffering conditions.

**Fig. 12:**
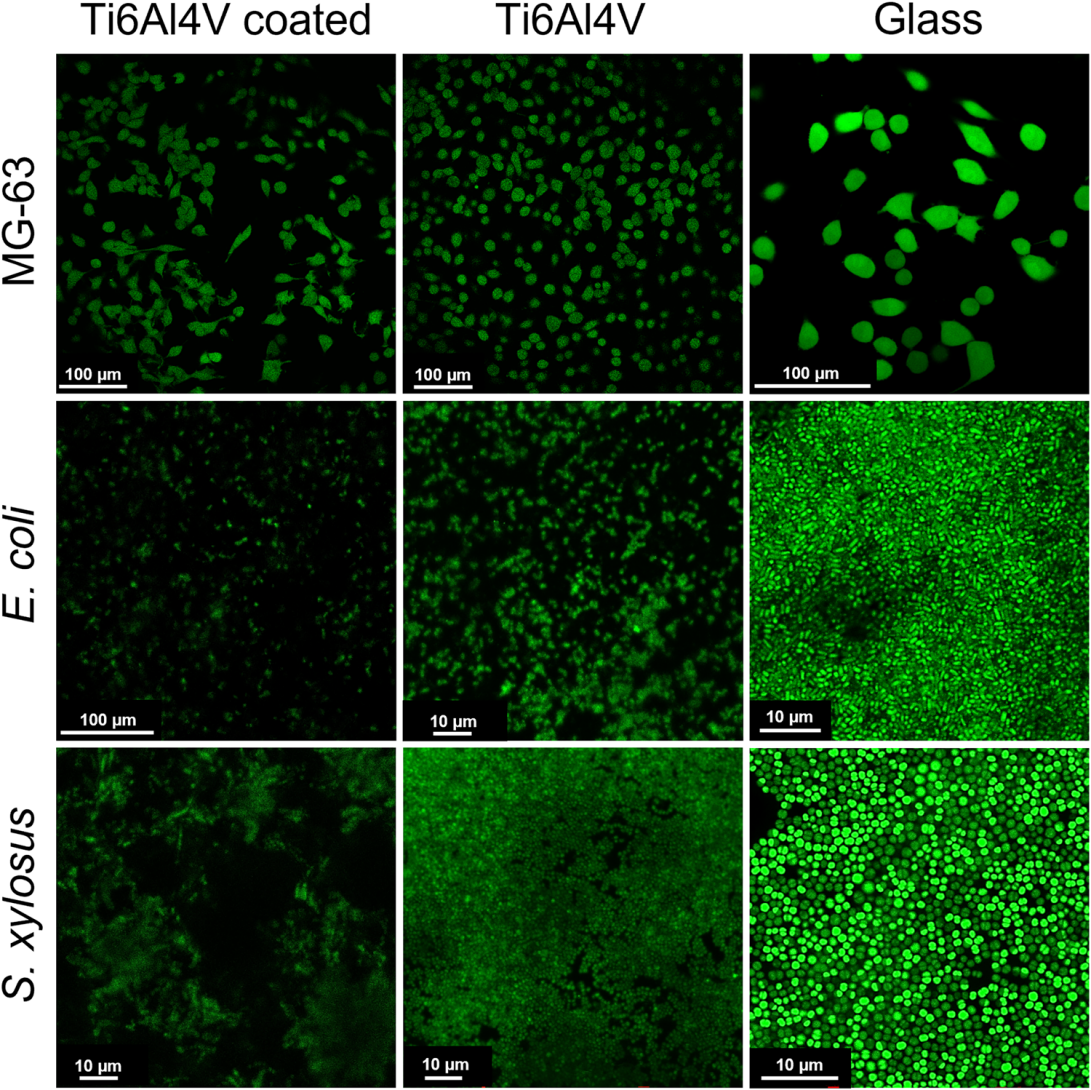
CLSM images of MG-63 cells grown for 48 h (**top row**), *E*. *coli* biofilm grown for 72 h (**centre row**), and *S*. *xylosus* biofilm grown for 72 h (**bottom row**) on calcium phosphate-coated Ti6Al4V, on uncoated Ti6Al4V, and on a glass substrate. Living cells are stained green

Confocal laser scanning microscopy showed a strong adherence of bacteria and cells to bare Ti6Al4V, to calcium phosphate-coated Ti6Al4V, and to glass (Fig. 12). There was no significant difference in the density of MG-63 cells between the three substrates. The density of the bacteria was very high on the glass substrate but less on the uncoated Ti6Al4V. Notably, even fewer bacteria were detected on calcium phosphate-coated Ti6Al4V. The optical resolution on the metal plates was lower than on glass because, in these cases, the microscope was operated in reflection mode and not in transmission mode. The confocal micrographs also indicated that the bacterial cells were present in smaller colonies, probably inside the porous coating, rather than forming a more confluent biofilm.

## Discussion

Calcium phosphate coatings on implant metals have been prepared earlier and studied in terms of structure, morphology, adhesion strength, and porosity [23, 36, 38, 40–42]. However, the effect of bacteria to form local biofilms on such coatings has not yet been studied. Two different model species that mimic bacteria with clinical relevance have been cultivated.

In general, *S*. *xylosus* produced significantly more biofilm mass than *E*. *coli*. This observation is consistent with the statistical analysis, which revealed a significant interaction between bacterial species and surface type, indicating that the effect of the substrate surface on biofilm formation differs between the two microorganisms. *E*. *coli* proliferated more strongly on the calcium phosphate-coated substrates than on the pure metals, probably due to the internal porosity of the calcium phosphate coating. These results suggest that the porous calcium phosphate coating particularly promotes biofilm formation by *E*. *coli*, whereas *S*. *xylosus* forms substantial biofilm biomass on both coated and uncoated surfaces. The different behavior of *E*. *coli* and *S*. *xylosus* on calcium phosphate-coated substrates may also be related to differences in bacterial shape and growth characteristics. The rod-shaped *E*. *coli* cells more easily access and colonize the narrow cavities within the porous coating structure, whereas the spherical morphology of *S*. *xylosus* leads to a different spatial organization within the biofilm. In addition, local microenvironments within the porous calcium phosphate structure may influence bacterial metabolism, for example, by localized retention of nutrients or metabolic products. Such effects may contribute to the increased biofilm biomass observed for *E*. *coli* on the coated metals. *S*. *xylosus* showed a strong biofilm formation on all substrates; only on pure titanium, the growth was slower (as with *E. coli*). This underscores the potential of different bacteria to colonize implant surfaces, more or less independent from the nature of the metal the presence of a coating with calcium phosphate.

Both bacterial species changed the pH of the culture medium, although this medium was buffered. The phenomenon of an increasing pH by *E*. *coli*, particularly intense during the first 24 h of cultivation, has also been reported by Thirstrup et al. [43]. It has been ascribed to the nutrient metabolism of *E*. *coli*, in particular, the consumption of amino acids and the subsequent release of ammonia (NH_3_) [44]. Although *E*. *coli* is a neutrophilic bacterium, it tolerates moderately alkaline conditions (up to pH 9) [45]. The acidification caused by *S*. *xylosus* is also due to the bacterial metabolism. *S. xylosus* is widely used in the food industry, e.g., for the production of fermented meat [46]. It forms acidic by-products (e.g., lactic acid, acetic acid), especially by carbohydrate metabolism [47]. Thus, the physiological properties of the two bacterial strains and the observed pH drop explain the corrosion of the acid-soluble calcium phosphate coating in the case of *S. xylosus*. As a rule, one of the most important factors that influences biofilm formation by bacteria is the local pH [48]. It affects biofilm establishment, formation and stability [49, 50]. On the other hand, the biofilm can also change the pH of its environment, e.g., by acidification as a result of the metabolization of nutrients, as we have shown here.

The calcium phosphate coating does not prevent the growth of bacteria and the formation of biofilms. In contrast, for *E. coli*, it enhanced bacterial adhesion, probably due to the presence of accessible cavities within the porous coating structure. It should also be noted that the crystal violet assay quantifies the total biofilm biomass, including bacterial cells, as well as extracellular polymeric substances (EPS). In the case of porous coatings, crystal violet may also be retained within cavities of the coating structure. Therefore, higher absorbance values in this assay may reflect the accumulation of biofilm biomass within the porous architecture rather than a higher density of bacterial cells directly visible on the outer surface in microscopic images.

Bacterial growth on implants will depend not only on the chemical nature of the coating (here: octacalcium phosphate) but also on its roughness and internal porosity. This has been demonstrated in the literature. The growth of *Staphylococcus epidermis* on pure TiO_2_ and on TiO_2_ coated with various types of nanoparticulate calcium phosphate did not show a significant difference between the uncoated and the coated substrates [51]. in good agreement with our results. After all, the surface of titanium and titanium alloys consists of TiO_2_ due to surface passivation [52]. In a meta-review, it was found that the biofilm formation was not dependent on the roughness of titanium surfaces [53]. However, the growth of bacteria inside a porous coating is obviously different from a two-dimensional surface with variable roughness. This distinguishes a calcium phosphate layer that is deposited from solution from similar layers prepared by other techniques like thermal plasma spraying [16], magnetron plasma sputtering [25], electrophoretic deposition [54], or microarc oxidation [55].

It should be noted that the present study considers biofilm formation by single bacterial species. In clinically relevant implant infections, biofilms are frequently polymicrobial, and interactions between different bacterial species may influence biofilm architecture, metabolic activity, and resistance to antimicrobial treatment. Therefore, while monospecies biofilms provide useful mechanistic insight into bacteria–material interactions, future studies should address how polymicrobial communities interact with porous calcium phosphate coatings.

## Conclusions

Typical implant materials can be well coated by octacalcium phosphate (OCP). The platelet-like deposited layer had a thickness of about 20 µm. MG-63 osteoblast-like cells grew well on the calcium phosphate layer, confirming its suitability to coat metallic implants in bone contact. However, both bacterial species studied here (*E*. *coli* and *S*. *xylosus*) also adhered well to calcium phosphate and migrated into cavities of the porous coating. The high bacterial biomass production on the calcium phosphate-coated samples also indicates the formation of biofilms associated with the porous coating, where they may be less accessible to antibiotics or mechanical removal. The surface-sensitive method, SEM, is complemented by confocal microscopy, which shows the local distribution of bacteria. Both bacterial species changed the local pH during 72 h cultivation, which led to acidic corrosion of the calcium phosphate layer in the case of *S. xylosus*. Depending on the implantation site, this may lead to enhanced degradation of this osteoconductive layer.

## Supplementary information

Supplementary information

## Data Availability

All data generated or analyzed during this study are included in this article.
