# Supplementary material for: Biofilm formation associated with calcium phosphate coating on implant metals
Source: J Mater Sci Mater Med. 2026 Apr 21;37(1):57. doi: 10.1007/s10856-026-07053-y (PMC13102785; doi:10.1007/s10856-026-07053-y)
Supplement: Supplementary file 1 — Supplementary information [file 10856_2026_7053_MOESM1_ESM.pdf]

Supporting information

Biofilm formation associated with calcium phosphate coating on implant metals

David Mrosek, Nataniel Białas, Aileen Winter, Oleg Prymak, Kateryna Loza, and Matthias Epple
Inorganic Chemistry and Center for Nanointegration Duisburg-Essen (CENIDE), University of Duisburg-Essen, 45141 Essen, Germany

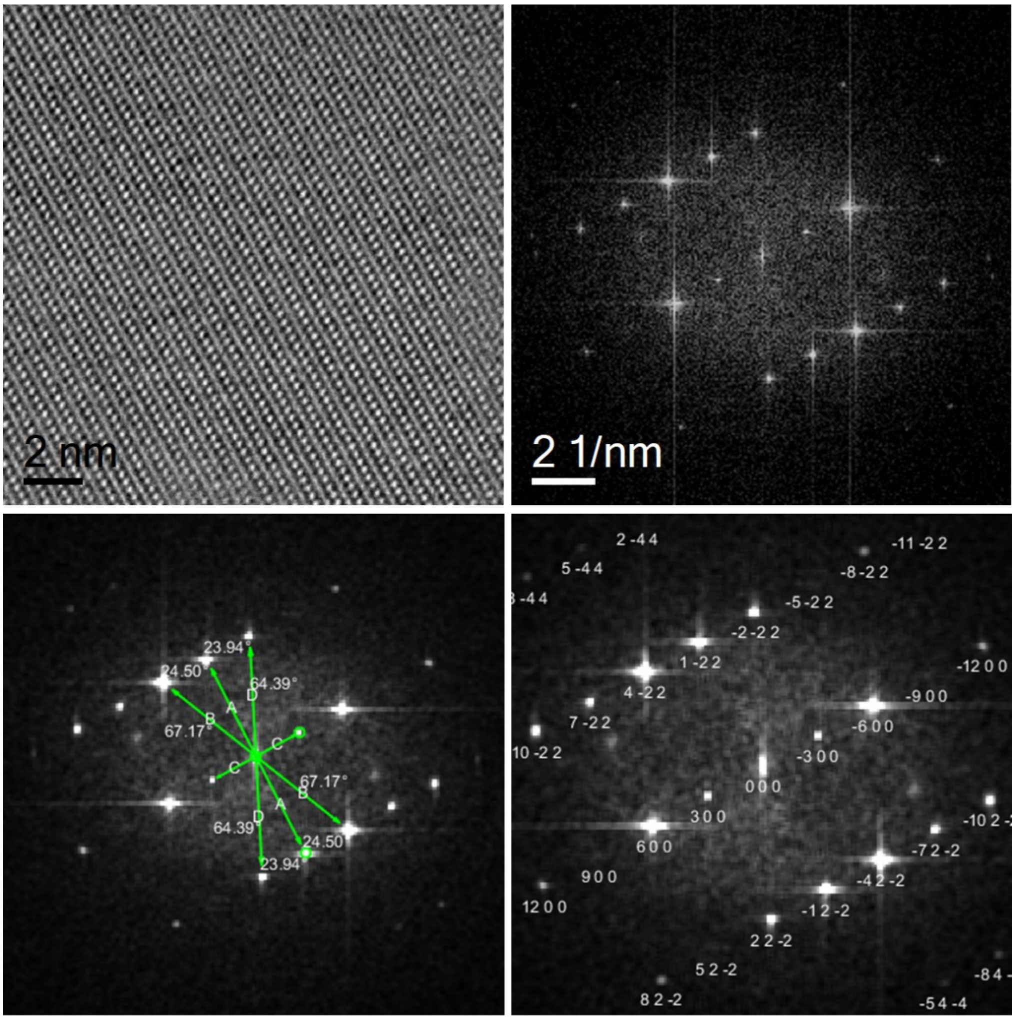

Vector identification	
A	-1 2 -2
B	-4 2 -2
C	-3 0 0
D	-2 -2 2

**Zone axis:**	0 1 1
**Estimation rating:**	Fair
**Consistency check:**	OK
**Lattice check:**	OK
**Total angular distance:**	3.24
**D-spacing STDEV:**	0.0048

Figure S1: High-resolution TEM (HRTEM) image of the calcium phosphate coating and the corresponding fast Fourier transform (FFT) image. The FFT shows discrete diffraction spots characteristic of a single-crystalline domain. The spots were indexed according to the reference pattern of octacalcium phosphate (ICSD 65347).

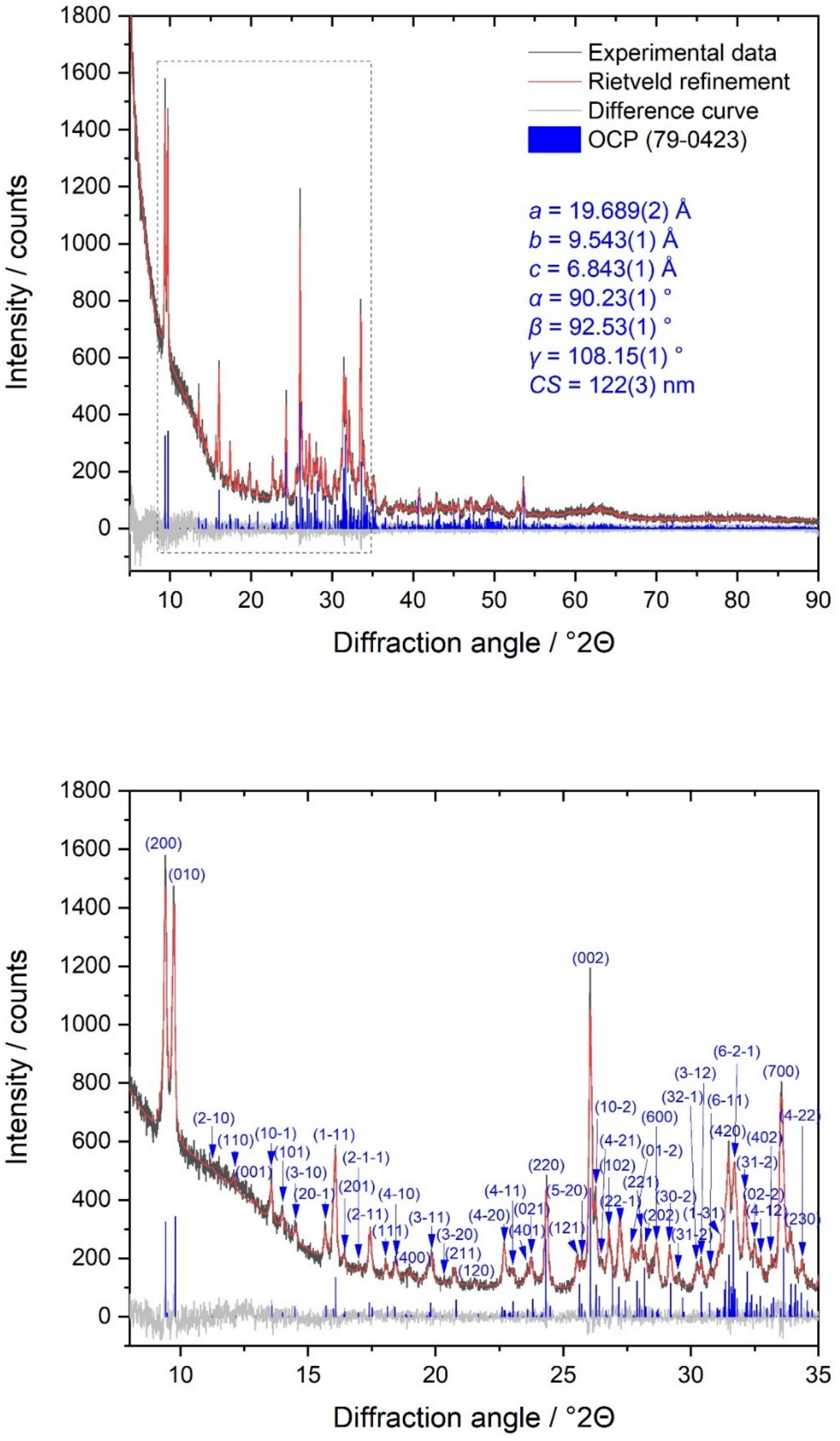

Figure S2: Rietveld refinement of OCP powder scraped off from 6 different coated titanium substrates (**top**) with a zoomed 2θ region from 8 to 35 ° for a better visualization of the OCP diffraction peaks with the corresponding Miller indices (**bottom**). *CS* denotes the crystallite size from Rietveld refinement. The diffractogram was measured in Bragg-Brentano mode.

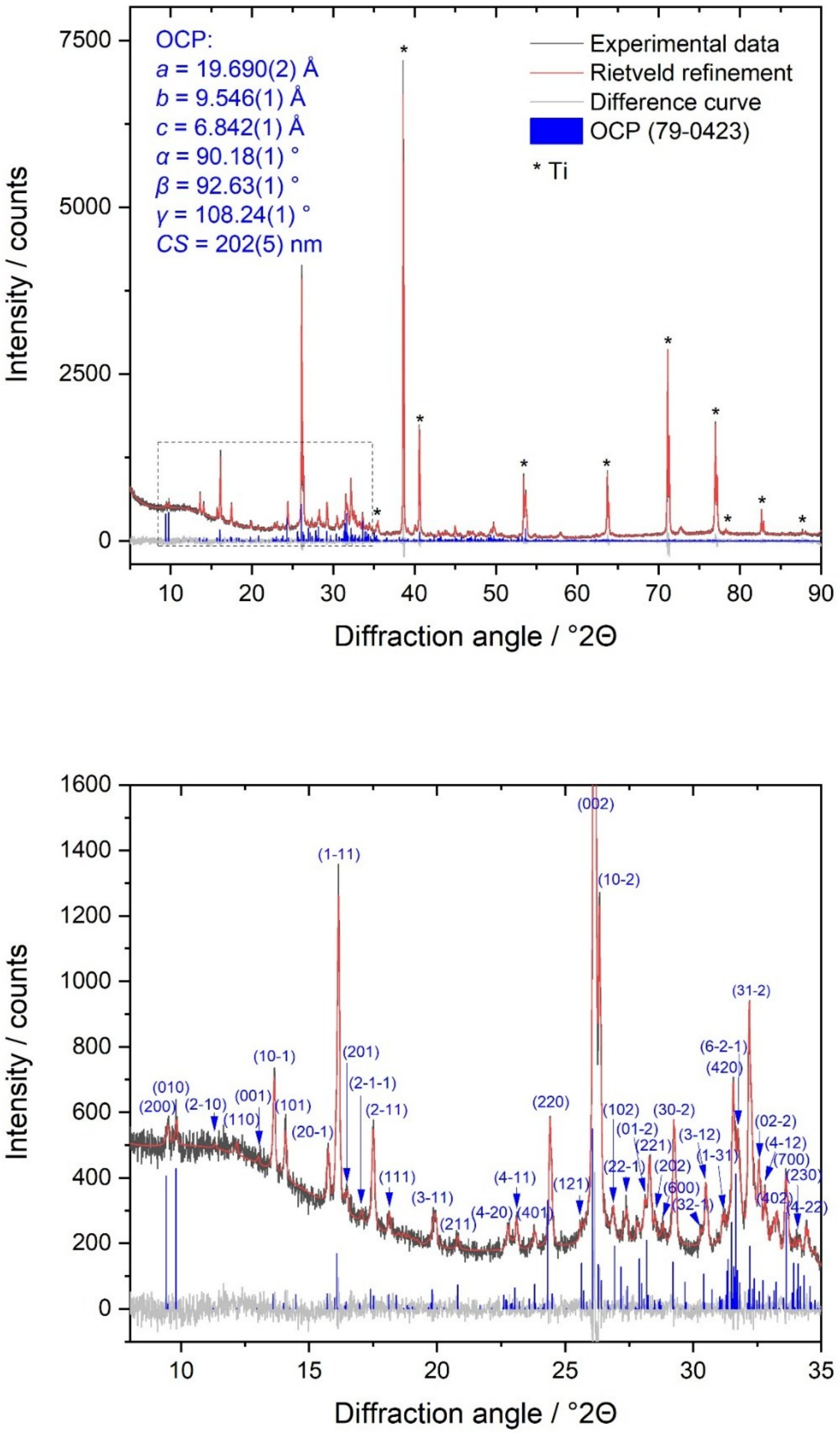

Figure S3: Rietveld refinement of OCP on Ti6Al4V (**top**) with a zoomed 2θ region from 8 to 35 ° for a better visualization of the OCP diffraction peaks with the Miller indices (**bottom**). The diffraction peaks of Ti are indicated by asterisks. The diffractogram was measured in Bragg-Brentano mode.

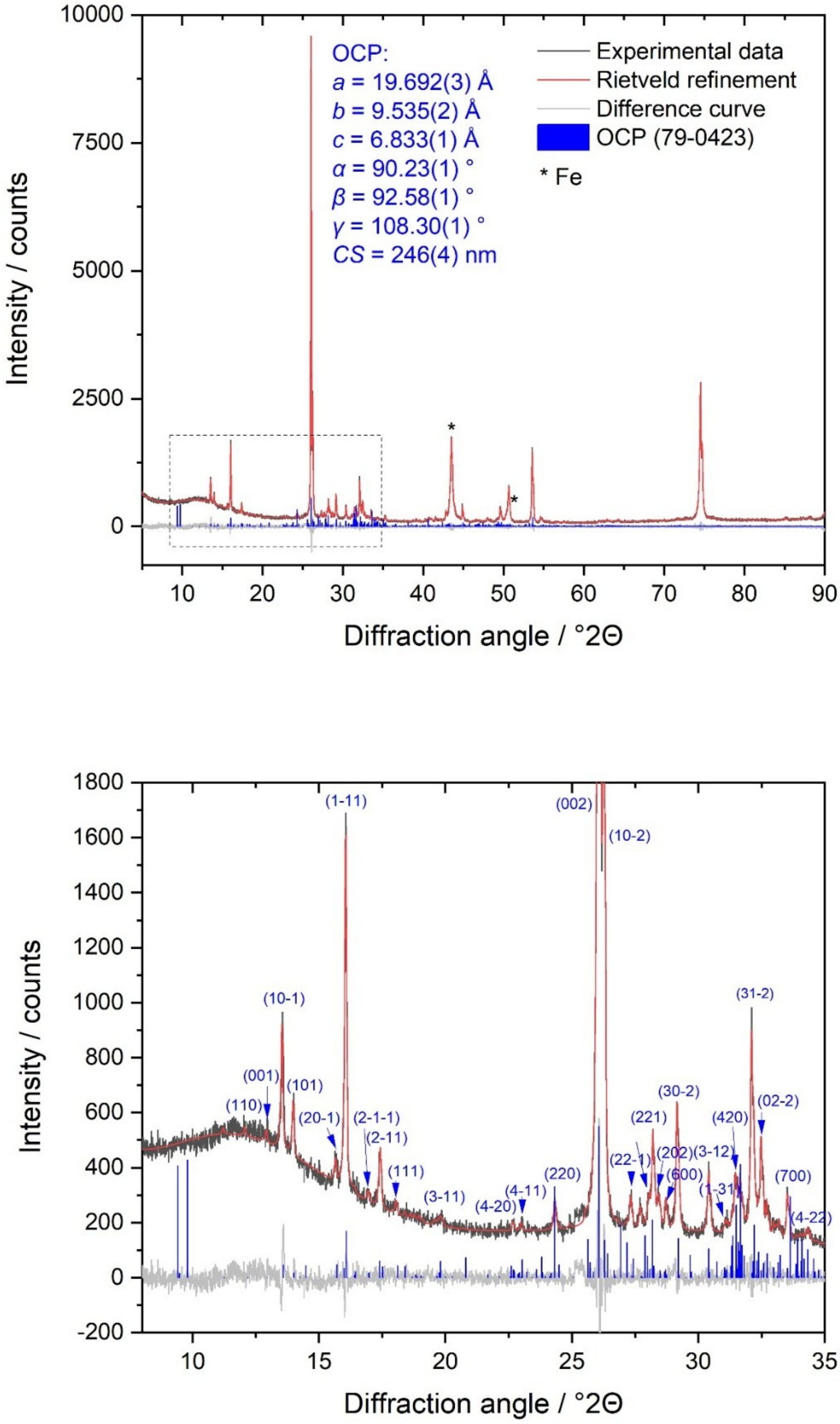

Figure S4: Rietveld refinement of OCP on 316L stainless steel (**top**) with a zoomed 2θ region from 8 to 35 ° for a better visualization of the OCP diffraction peaks with the Miller indices (**bottom**). The diffraction peaks of Fe are indicated by asterisks. The diffractogram was measured in Bragg-Brentano mode.
